# DIAPH1 Is Upregulated and Inhibits Cell Apoptosis through ATR/p53/Caspase-3 Signaling Pathway in Laryngeal Squamous Cell Carcinoma

**DOI:** 10.1155/2019/6716472

**Published:** 2019-01-14

**Authors:** Jiechao Yang, Liang Zhou, Yanping Zhang, Juan Zheng, Jian Zhou, Zheqiang Wei, Jiaping Zou

**Affiliations:** ^1^Department of Otorhinolaryngology-Head and Neck Surgery, The Third People's Hospital, Affiliated Hospital of Jiangnan University, Wuxi, Jiangsu, China; ^2^Department of Otorhinolaryngology-Head and Neck Surgery, Eye, Ear, Nose and Throat Hospital, Fudan University, Shanghai, China; ^3^Laboratory of Eye, Ear, Nose and Throat Hospital, Fudan University, Shanghai, China; ^4^Department of Pathology, The Third People's Hospital, Affiliated Hospital of Jiangnan University, Wuxi, Jiangsu, China

## Abstract

Cancer bioinformatics has been used to screen possible key cancer genes and pathways. Here, through bioinformatics analysis, we found that high expression of diaphanous related formin 1 (DIAPH1) was associated with poor overall survival in head and neck squamous cell carcinoma and laryngeal squamous cell carcinoma (LSCC). The effect of DIAPH1 in LSCC has not been previously investigated. Therefore, we evaluated the expression, function, and molecular mechanisms of DIAPH1 in LSCC. Immunohistochemistry and western blot analysis confirmed the significant upregulation of DIAPH1 in LSCC. We used DIAPH1 RNA interference to construct two DIAPH1-knockdown LSCC cell lines, AMC-HN-8 and FD-LSC-1, and validated the knockdown efficiency. Flow cytometry data showed that DIAPH1 inhibited apoptosis. Further, western blot analysis revealed that DIAPH1 knockdown increased the protein levels of ATR, p-p53, Bax, and cleaved caspase-3, -8, and -9. Thus, DIAPH1 is upregulated in LSCC and may act as an oncogene by inhibiting apoptosis through the ATR/p53/caspase-3 pathway in LSCC cells.

## 1. Introduction

Laryngeal carcinoma is a common malignant tumor of the head and neck region. It is estimated that worldwide there were 156,877 cases of laryngeal carcinoma in 2012, accounting for 1% of all cancers and 20,014 cases in China. In 2012 an estimated 1% of cancer deaths were due to laryngeal carcinoma, which equates to 83,376 deaths worldwide, 12,308 of which were in China [1]. It was reported that in the United States, there were 13,430 new cases of laryngeal carcinoma and 3620 cancer deaths in 2016 [2]. Laryngeal squamous cell carcinoma (LSCC) accounts for more than 95% of laryngeal carcinoma [3]. Surgery and chemoradiotherapy are the primary treatments for LSCC. However, the five-year survival rate and quality of life, especially for advanced patients, have not improved in recent years [2]. Thus, there is an urgent need to find new diagnostic biomarkers or new treatments that target LSCC.

Cancer bioinformatics has emerged as a useful tool to assist researchers in screening through databases to identify potential key cancer genes and pathways. Further experiments and clinical studies would be used to verify the accuracy of such analyses. Analysis of the data from 262 patients with head and neck cancer from the Cancer Genome Atlas (TCGA, https://gdc.cancer.gov/), diaphanous related formin 1 (DIAPH1), also termed mDia1, is considered a significant independent prognostic factor for overall survival (OS). Further experiments verify that DIAPH1 is involved in the wound healing and the metastasis of head and neck squamous cell carcinoma (HNSCC) [4].

Apoptosis is an inbuilt cellular mechanism used to remove harmful cells, including malignant cells, from bodies. Abnormal apoptosis is closely related to tumor initiation and progression [5]. It was reported that knockdown of DIAPH1 in human U87 glioblastoma cells could significantly promote cell apoptosis [6]. In the development process of anemia and ineffective erythropoiesis in mice with mDia1/miR-146a double knockout, caspase-3 activation and apoptosis played a key role [7]. Therefore, we hypothesized that, in LSCC, a type of HNSCC, DIAPH1 may also influence tumorigenesis and tumor development through interfering with the apoptosis of tumor cells. The role of DIAPH1 in apoptosis in LSCC has not previously been investigated.

In this study, bioinformatics analyses, verification of clinical samples, and apoptosis-related experiments to partially delineate the role of DIAPH1 in apoptosis in LSCC.

## 2. Materials and Methods

### 2.1. Bioinformatics Analysis

Bioinformatics analysis of the data from the cBioPortal for cancer genomics (http://www.cbioportal.org/) was performed to investigate the clinical features of DIAPH family members in HNSCC. The data from the TCGA was used for survival analysis based on the differentiation degree. According to the differentiation grade, the patients were divided into class G1 (low grade), G2 (intermediate grade), and G3 (high grade). DIAPH1 expression difference between tumor and adjacent normal tissues was also examined. In addition, the GSE65858 dataset was downloaded from Gene Expression Omnibus (GEO, https://www.ncbi.nlm.nih.gov/geo/) and used for survival analysis based on the tumor site. In survival analysis, the patients were divided into high and low expression groups based on median DIAPH1 mRNA expression levels.

### 2.2. LSCC Patients and Tissue Sample Collection

LSCC tissues and matching adjacent nontumor (ANT) tissues were collected from thirty-six LSCC patients undergoing total or partial laryngectomy at the Department of Otorhinolaryngology-Head and Neck Surgery of the Eye and ENT Hospital of Fudan University between April and September 2016. No patients had undergone chemoradiotherapy or other biological therapies before surgery. All tissue samples were collected in accordance with the study protocols approved by the Ethics Committee of Fudan University, and all subjects provided written informed consent prior to being added to the study. All patients were classified according to the American Joint Committee on Cancer guidelines, and the correlation between the DIAPH1 expression level and clinicopathological characteristics was analyzed for all patients.

### 2.3. Immunohistochemistry (IHC)

For IHC, the tissue samples were fixed in 10% neutral buffered formalin (Weigesi Biotech, China) and embedded in paraffin. Embedded tissues were then cut into 4 mm sections. After a series of procedures including deparaffinization, gradient dehydration, antigen retrieval, blocking of endogenous peroxidase activity, and blocking nonspecific binding, the slides were incubated with anti-DIAPH1 antibody (1 : 150; Abcam, UK, cat. no. ab129167) at 4°C overnight. Then, the ChemMate™ Envision™ Detection Kit was used according to the manufacturer's instructions (Gene Tech, China, cat. no. GK500710). This involved the application of a secondary antibody to the sections at 37°C for 0.5 hours, followed by diaminobenzidine staining. Two pathologists, blinded to the sample type, independently scored IHC staining. We used an immunohistochemical score (IHS) value to estimate the DIAPH1 expression level, as previously described [8].

### 2.4. Cell Lines and Cell Culture

Two LSCC cell lines, AMC-HN-8 and FD-LSC-1, were used in this study. AMC-HN-8 cells were obtained from the Cell Bank of the Chinese Institute of Biochemistry and Cell Biology (Shanghai, China) and cultured in RPMI-1640 (Gibco, USA) containing 10% fetal bovine serum (FBS, Gibco, USA), 1% penicillin and streptomycin (Genom, Hangzhou, China), and 1% L-glutamine (Genom) in a 5% CO_2_ incubator with humidified air. FD-LSC-1 cells were obtained from our lab [9] and cultured in BEGM™ Bronchial Epithelial Cell Growth Medium (Lonza, Walkersville, MD, USA), supplemented with 10% FBS (Gibco), 1% penicillin and streptomycin (Genom), and 1% L-glutamine (Genom) in a 5% CO_2_ incubator with humidified air.

### 2.5. Construction of DIAPH1 RNA Interference Vectors and Lentiviral Infection

Four short hairpin RNA (shRNA) sequences targeting DIAPH1 gene were designed for DIAPH1 knockdown (Table 1). The recombinant DIAPH1-shRNA lentivirus (SH1, SH2, SH3, and SH4) and the negative control lentivirus (NC), all carrying a green fluorescent protein (GFP) sequence, were purchased from Hanyin Co (Shanghai, China). AMC-HN-8 cells were infected with five successfully constructed lentivirus vectors, and puromycin-resistant cell clones were screened using puromycin. Stable DIAPH1 knockdown (SH1, SH2, SH3, and SH4) and negative control (NC) cell lines were then obtained. Furthermore, FD-LSC-1 cells were infected with SH1, SH2, and NC lentivirus, and the puromycin-resistant cell clones were screened using puromycin to obtain stable FD-LSC-1 SH1, SH2, and NC cell lines.

### 2.6. Quantitative Reverse Transcription PCR (RT-qPCR)

Total RNA was extracted from AMC-HN-8 SH1, SH2, SH3, SH4, and NC cells using the RNA Extraction Reagent Kit (CWBiotech, China) in accordance with the manufacturer's instructions. Total RNA was reverse transcribed into cDNA using the PrimeScript RT Reagent Kit (Takara, Japan). Subsequently, qPCR was performed using SYBR Primix Ex Taq (Takara, Japan) and an RT-qPCR system (ABI PRISM 7500, Applied Biosystems, CA), which was also used to analyze the data. Recombinant ribosomal protein L13A (RPL13A) was chosen as the internal control. The 2^-ΔΔCt^ method was used to calculate relative mRNA expression (DIAPH1/RPL13A). The same method was applied to FD-LSC-1 SH1, SH2, and NC cells using glyceraldehyde-3-phosphate dehydrogenase (GAPDH) as the internal control. All primers used for RT-qPCR were designed and synthesized by Hanyin Co (Shanghai, China). The primers used for RT-qPCR were shown in Table 2.

### 2.7. Western Blot Analysis

Proteins were harvested from the cell groups (described above) and tissue samples from seven LSCC patients. The RIPA protein extraction reagent (Beyotime, Shanghai, China), containing a protease inhibitor cocktail (Weiao Biaotec, Shanghai, China), was used to extract total proteins, and the supernatants were collected following centrifugation at 4°C. The BCA protein assay (Beyotime) was used to measure protein concentration. Equal quantities of protein were separated via 10% SDS-polyacrylamide gel electrophoresis and transferred onto polyvinylidene fluoride membranes (PVDF; Millipore Corporation, Bedford, MA, USA). The PVDF membranes were blocked with 5% skim milk in TBST (Sangon Biotech, Shanghai, China) for 1 hour at room temperature (RT). The membranes were incubated with primary antibodies overnight at 4°C and then with horseradish peroxidase-conjugated secondary antibodies (1 : 2000, Jackson ImmunoResearch, USA) for 2 hours at RT. The primary antibodies used were anti-DIAPH1 (1 : 1000, Abcam, cat. no. ab129167); anti-ataxia-telangiectasia-and Rad3-related (ATR 1 : 500, Santa Cruz Biotechnology [Santa], USA, cat. no. sc-515173); anti-phospho-p53 (p-p53 1 : 1000, Cell Signaling Technology [CST], USA, cat. no. 9286); anti-BCL2 associated X (Bax 1 : 1000, CST, cat. no. 5023); anti-cleaved caspase-3 (1 : 1000, CST, cat. no. 9664); anti-cleaved caspase-8 (1 : 500, Santa, cat. no. sc-56070); anti-cleaved caspase-9 (1 : 500, Santa, cat. no. 8355); and anti-GAPDH (1 : 1000, Abcam, cat. no. AC002). Finally, enhanced chemiluminescence reagent (ECL Kit, Beyotime) and autoradiography was used to visualize the protein bands (Carestream Health, Canada).

### 2.8. Apoptosis Analysis

Apoptosis analysis was performed for two DIAPH1 RNA-interference groups and one control group. The three groups of cells were simultaneously planted into 6 cm cell culture dishes and were collected once they reached 80% confluence. Following the protocol of the Phycoerythrin (PE) Annexin V Apoptosis Detection Kit (BD PharMingen, CA, USA), we used cold phosphate buffer saline (PBS) to wash cells and added 1x Binding Buffer to form a single-cell suspension. After staining with PE Annexin V and/or 7-AAD, these tubes of cell suspension were analyzed with flow cytometry (FCM, BD FACSCalibur) within one hour. The same method was applied to AMC-HN-8 and FD-LSC-1 cells.

### 2.9. Statistical Analysis

In the bioinformatics analysis, RNA sequencing FPKM-UQ values from the mRNA were used as a measure of the expression level of genes. The log-rank test and the R platform (v 3.4.1) were used in survival analyses. Except for bioinformatics analyses, all data are presented as the mean (± standard deviation, SD), and all statistical analyses were conducted with SPSS 22.0 statistical software (IBM, Armonk, NY, USA). The differences between groups were compared using the Mann-Whitney *U* test and the Student's *t*-test, and a two-tailed *p* < 0.05 was considered statistically significant.

## 3. Results

### 3.1. High DIAPH1 Expression Is Associated with Poor Overall Survival of Patients with HNSCC

**Figure 1:**
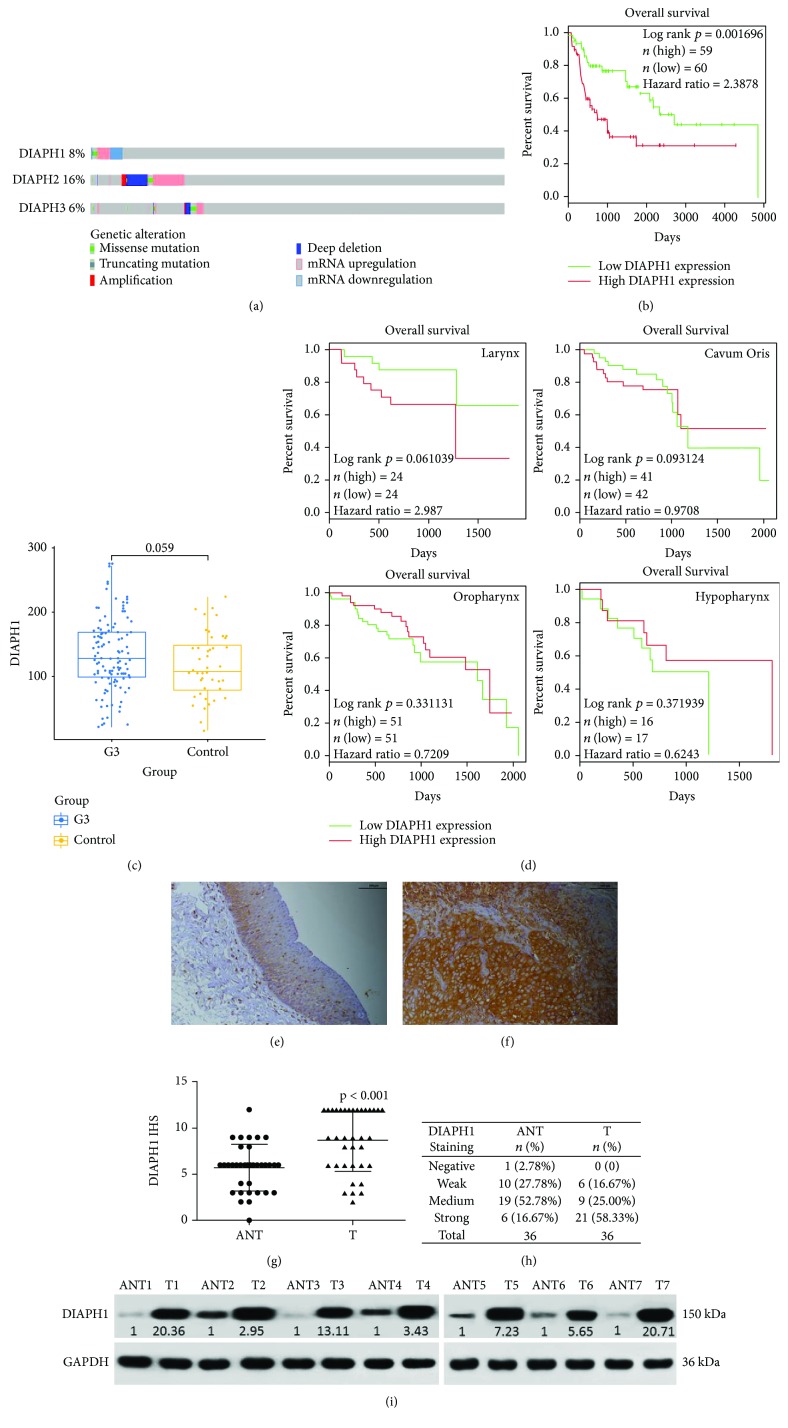
Bioinformatics and immunohistochemical score (IHS) analysis of DIAPH1. (a) Expression levels of DIAPH1-3 in HNSCC from the TCGA dataset. (b) High expression of DIAPH1 was significantly associated with poor OS in class G3 HNSCC as determined by log-rank test and R platform from TCGA dataset (*p* = 0.002, hazard ratio = 2.39). (c) DIAPH1 expression was higher in G3 HNSCC tissues than in normal tissues as determined by the Student's *t*-test from TCGA dataset (*p* = 0.059). (d) High expression of DIAPH1 was associated with poor OS in LSCC as determined by log-rank test and R platform from GSE65858 dataset (*p* = 0.061, hazard ratio = 2.99). (e) Weak expression level of DIAPH1 in adjacent nontumor (ANT) tissues (scale bar 100 *μ*m). (f) Strong expression level of DIAPH1 in primary LSCC (T) tissues (scale bar 100 *μ*m). (g) IHS values were calculated using the Mann-Whitney *U* test. (h) Representative expression of DIAPH1 by IHS in ANT and T tissues. (i) Western blot analysis of DIAPH1 protein expression in seven paired ANT and T tissues.

First, we analyzed the data of 530 patients with HNSCC (TCGA, Provisional, http://www.cbioportal.org/) and found that abnormal mRNA expression and DIAPH1, DIAPH2, and DIAPH3 mutations were frequently identified in HNSCC (Figure 1(a)). Second, we analyzed clinical and mRNA expression data of 500 patients with HNSCC, from TCGA, and found that in class G3, high DIAPH1 expression was significantly associated with poor OS (*p* = 0.002, Figure 1(b)) and that the expression of DIAPH1 was higher in tumor tissues than in adjacent normal tissues (Figure 1(c)). However, we did not find significant association in class G1 and G2, which may mean DIAPH1 overexpression and poor prognosis are associated only in high-grade carcinomas. Moreover, because different types of HNSCCs, such as LSCC and hypopharyngeal squamous cell carcinoma, have distinctively different biologic behaviours, we analysed GSE65858 dataset based on different primary sites and found the trend that high DIAPH1 expression was associated with poor OS in LSCC (Figure 1(d)).

### 3.2. DIAPH1 Is Upregulated in LSCC

IHC analysis of 36 paired LSCC and ANT tissues showed DIAPH1 protein expression was significantly upregulated in LSCC tissues (Figures 1(e)–1(h)). LSCC and matching ANT tissues from seven patients whose IHS of LSCC tissues were higher than those of their ANT tissues and whose clinical stages were III or IV were used for western blot analysis. The western blot results showed that DIAPH1 is upregulated in LSCC (Figure 1(i)). Furthermore, the DIAPH1 expression was significantly higher in clinical stages III and IV than in clinical stages I and II (*p* = 0.016, Table 3).

### 3.3. Construction of DIAPH1-Knockdown Cells and Determination of Knockdown Efficiency

**Figure 2:**
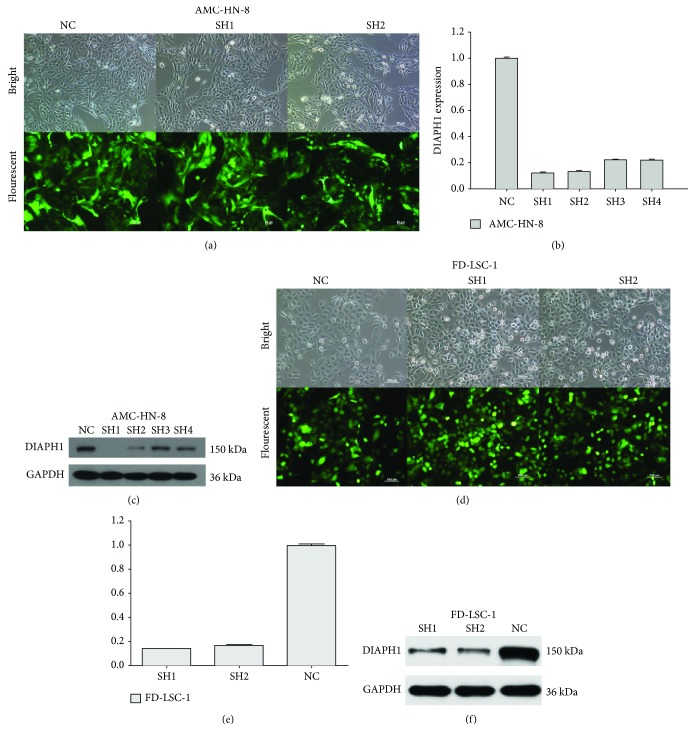
DIAPH1 knockdown efficiency by shRNA lentivirus (SH). (a, d) Bright and fluorescent photomicrographs were taken 72 hours after lentivirus infection of DIAPH1 knockdown (SH) and the negative control (NC) in AMC-HN-8 cells (scale bars 50 *μ*m) and FD-LSC-1 cells (scale bars 100 *μ*m). (b, e) Identification of DIAPH1 knockdown efficiency in DIAPH1 SH and NC cells of the abovementioned cells by RT-qPCR. (c, f) Western blot analysis of DIAPH1 protein expression in the abovementioned cells.

Stably transfected cells were successfully constructed and the proportions of cells that presented green fluorescence were over 80% (Figures 2(a) and 2(d)). RT-qPCR results suggested that DIAPH1 mRNA levels in SH1 and SH2 groups reduced more than 80% compared with that of the NC group (Figures 2(b) and 2(e)). Western blot analysis further verified the knockdown efficiency in SH1 and SH2 groups (Figures 2(c) and 2(f)). Taken together, these results show that, compared to SH3 and SH4, SH1 and SH2 efficiently knocked-down DIAPH1 expression.

### 3.4. Knockdown of DIAPH1 Promotes Apoptosis in AMC-HN-8 and FD-LSC-1 Cells

**Figure 3:**
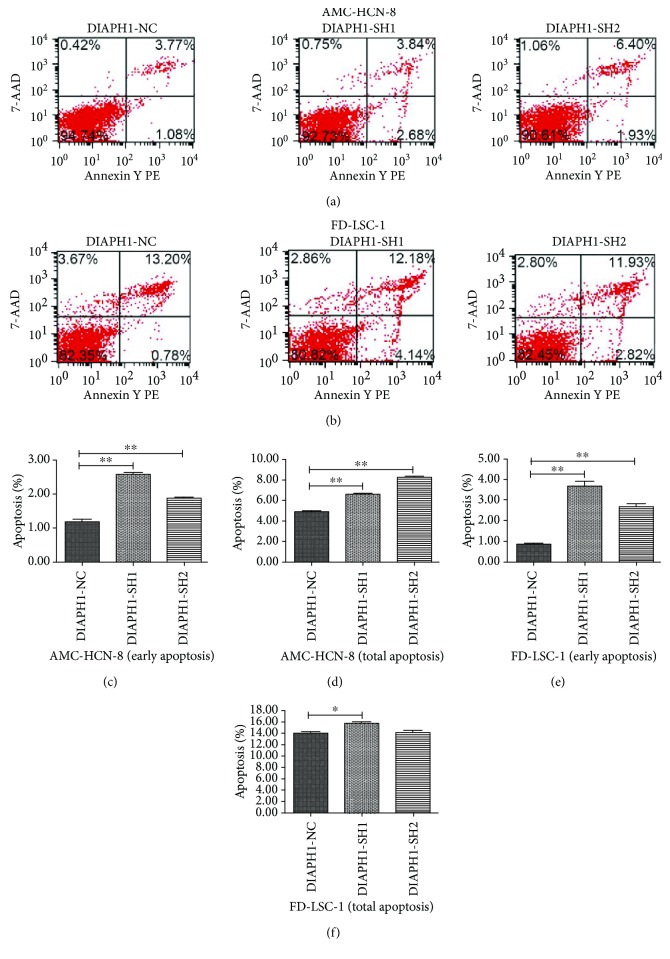
Apoptosis rate of DIAPH1 knockdown (SH) and the negative control (NC) in AMC-HN-8 and FD-LSC-1 cells as determined by flow cytometric analysis. (a, b) Representative graphs of apoptosis by flow cytometric analysis. (c, d) Early and total apoptosis in AMC-HN-8 SH1 and SH2 cells increased. (e) Early apoptosis in FD-LSC-1 SH1 and SH2 cells increased. (f) Total apoptosis in FD-LSC-1 SH1cells increased. ^∗∗^ *p* < 0.01, ^∗^ *p* < 0.05.

We used flow cytometry and costaining with PE Annexin V and/or 7-AAD to evaluate the effect of DIAPH1 knockdown on apoptosis in AMC-HN-8 and FD-LSC-1 cells (Figures 3(a) and 3(b)). In AMC-HN-8 cells, the proportion of cells in early apoptosis (PE Annexin V positive/7-AAD negative) was significantly higher in SH1 (2.58 ± 0.10%) and SH2 (1.88 ± 0.06%) cells than in NC (1.17 ± 0.14%) cells (*p* < 0.01 for both comparisons, Figure 3(c)). The level of total apoptosis (PE Annexin V positive, including apoptotic and dead cells) in SH1 (6.62 ± 0.24%) and SH2 (8.27 ± 0.10%) AMC-HN-8 cells was also significantly greater than that observed in NC (4.91 ± 0.09%) cells (*p* < 0.01 for both comparisons, Figure 3(d)). We repeated this analysis in FD-LSC-1 cells and found that, compared to the control (0.84 ± 0.07%), early apoptosis was increased in both SH1 (3.69 ± 0.39%) and SH2 (2.68 ± 0.24%) cells (*p* < 0.01 for both comparisons, Figure 3(e)). However, when analyzing the total level of apoptosis in the same cells, only SH1 (15.73 ± 0.57%) cells showed results significantly different to those of the controls (SH2, 14.08 ± 0.79%; NC, 14.02 ± 0.48%; SH1 vs. NC, *p* = 0.017, Figure 3(f)). Taken together, these results suggest that DIAPH1 knockdown promotes early apoptosis.

### 3.5. DIAPH1 Resisted Apoptosis via ATR/p53/Caspase-3 Signaling Pathway

**Figure 4:**
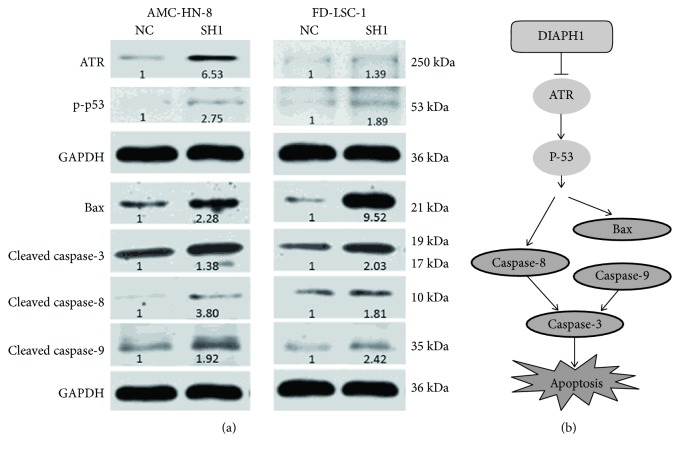
DIAPH1 knockdown promoted apoptosis via ATR/p53/caspase-3 signaling pathway. (a) Western blot analysis of ATR, p-p53, Bax, and cleaved caspase-3, -8, and -9 in DIAPH1 knockdown (SH1) and control (NC) of AMC-HN-8 and FD-LSC-1 cells. Numbers labelled under the bands were the relative expression and the relative expression of NC cells was set as 1. (b) The schematic graph of apoptotic signaling pathway mediated by DIAPH1.

To further investigate the mechanisms by which DIAPH1 functions to resist apoptosis, we performed western blotting for genes associated with the ATR/p53/caspase-3 signaling pathway. We found that DIAPH1 knockdown results in an upregulation of ATR, p-p53 (Ser 15), Bax, and cleaved caspase-3, -8, and -9 protein expression (Figure 4(a)).

## 4. Discussion

The DIAPH1 gene was initially identified in mammalian cells by Watanabe et al. in 1997 [10]. DIAPH1 belongs to the formin family and has two functional domains: the formin-homology 1 and 2 domains (FH1 and FH2) and several protein regulatory domains, such as the GTPase-binding domain and the dimerization domain [11]. Previous research has shown that, using the FH1 and FH2 domains, DIAPH1 can nucleate actin filaments [12]. DIAPH1 interacts with CLIP-170, actin-capping protein, or other associated proteins to accelerate actin filament elongation and regulate microtubule stabilization and the cytoskeletal system, to ultimately impact cellular functions, including cell migration and division [13–15]. Although the biological functions of DIAPH1 have been widely studied, its biological and clinical significance in cancer, especially in LSCC, remains poorly understood.

In the present study, bioinformatics analysis showed that in class G3 HNSCC, the expression of DIAPH1 was upregulated and high levels of DIAPH1 expression were significantly associated with poor OS. Moreover, high expression of DIAPH1 was associated with poor OS in LSCC. Consistent with the DIAPH1 expression patterns observed in glioma, colorectal and breast cancers, and oral squamous cell carcinoma [6, 16–18], IHC and western blotting of paired LSCC and ANT tissues showed that DIAH1 was overexpressed in LSCC. Furthermore, analysis of the correlation between DIAPH1 expression level and clinical variables showed that high DIAPH1 expression was significantly associated with increased clinical stage, which could result in a poor prognosis. These results imply that DIAPH1 may play a carcinogenic role in LSCC.

Previous researchers have shown that regulation of apoptosis is involved in tumor progression and that inhibitors of apoptosis are considered a promising adjuvant therapy of cancer. During apoptosis, a series of morphologic changes take place in cells. These changes include cell shrinkage, cell membrane blebbing, convolution of the nucleus, and dynamic remodeling of the cytoskeleton [19]. As regulator of the cytoskeletal system, DIAPH1 inevitably participates in the regulation of apoptosis. Lee et al. reported that mDia1 expression was upregulated in NADPH oxidase- (Nox1-) deficient cells. Moreover, Nox1 knockouts displayed reduced postinjury apoptosis, supporting the contention that mDia1 upregulation may reduce apoptosis [20]. Indeed, it was reported that mDia1 activation via the FH2 domain has been closely related to apoptosis [21]. To investigate the function of DIAPH1 in LSCC, especially in the apoptotic machinery, we constructed DIAPH1-knockdown cells using RNA interference technology and verified the knockdown efficiency of SH1 and SH2. Our flow cytometry analysis revealed that DIAPH1 could inhibit apoptosis in both AMC-HN-8 and FD-LSC-1 cells. The observed effect on apoptosis was greater in SH1 cells than in SH2 cells. Therefore, we chose to further investigate apoptotic mechanisms in AMC-HN-8 NC, AMC-HN-8 SH1, FD-LSC-1 NC, and FD-LSC-1 SH1 cells. RNA-sequencing analysis of AMC-HN-8 NC and SH1 cells showed that, compared with NC cells, SH1 cells expressed lower levels of ATR mRNA (fold change = 0.44, *p* = 5.52 × 10^−9^), and KEGG pathway analysis showed that the p53 signaling pathway was critical (data not shown).

DNA damage plays a critical role in tumor initiation and progression and severe DNA damage, such as DNA double-strand break (DSB) results in apoptosis. As the DNA damage-inducing apoptotic factor, ATR is the important checkpoint of the regulators upstream from the DNA damage pathway [19]. The p53 tumor suppressor inhibits tumorigenesis, and activation of the p53 pathway is involved in DNA damage repair and apoptosis. In response to DNA damage, p53 becomes phosphorylated and ATR mediates the phosphorylation of p53 on serine 15 (Ser 15) [22–24]. Here, DIAPH1 knockdown increased ATR and p-p53 protein levels in both AMC-HN-8 and FD-LSC-1 cells, implicating DIAPH1 in apoptosis.

Distinct but congregating intrinsic and extrinsic pathways mediate apoptosis through the chronological activation of caspases. DNA damage initiates the intrinsic pathway, which is controlled by BCL-2 family members and proapoptotic effectors, including BAX and BAK. These proapoptotic effectors cause the disruption of the mitochondrial membrane and the formation of the apoptosome, consisting of cytochrome c, Apaf-1, and caspase-9 [5, 25]. Cui et al. demonstrated oridonin-induced MCF-7 human breast cancer cell apoptosis was mediated by p53 through a caspase-9-dependent pathway. After treatment with oridonin, p-p53 and BAX proteins were upregulated, and activated caspase-9 was increased [26]. Our results showed that DIAPH1 downregulation elevates the expression of BAX and cleaved caspase-9, suggesting that DIAPH1 participates in the intrinsic apoptotic pathway.

The extrinsic apoptotic pathway is activated when death receptors tie up their ligands, such as FAS and tumor necrosis factor-related (TNFR) apoptosis-inducing ligands. This activates caspase-8 via the Fas-associated and TNFR-associated death domains [5, 25], which in turn activate caspase-3 and -7 [27]. In addition, the apoptosome formed in the intrinsic pathway activates the downstream caspase-9/3 signaling cascade [28]. Therefore, caspase-3 is an effector caspase of both intrinsic and extrinsic apoptotic pathways [5, 25]. It has been demonstrated that PP-22, a monomer isolated from the plant Paris polyphylla, exhibited anticancer activity toward human tongue squamous cell carcinoma SCC-15 cells. After the PP-22 treatment, the apoptotic rate of SCC-15 cells was significantly elevated and the caspase-8/3 signaling cascade was activated [29]. The same caspase-8/3 pathway activation occurred in DU-145 human prostate cancer cell apoptosis and was induced by the TNFR-associated factor 2 [30]. Choi et al. reported that indole-3-carbionol (I3C) plays an antitumor role in human lung cancer via an apoptosis-inducing effect. I3C induced p53 Ser 15 phosphorylation and the caspase-8/3 pathway [22]. The activation of the ATR/p53/caspase-3 signaling cascade in apoptosis has also been demonstrated [24]. Here, compared with NC cells, both AMC-HN-8 and FD-LSC-1 SH1 cells showed higher caspase-8/3 pathway activity, indicating that DIAPH1 knockdown activates the extrinsic apoptotic pathway mediated by ATR/p53/caspase-8/3.

Therefore, our study demonstrates that DIAPH1 knockdown promotes apoptosis via the ATR/p53/caspase-3 signaling in both the intrinsic and extrinsic apoptotic pathways (Figure 4(b)).

In this study contains some limitations. First, because of the high proportion of partial laryngectomies, the number of paired clinical tissue samples collected was not large. Second, in addition to apoptosis, p53 can regulate the cell cycle, so further studies examining the effect of DIAPH1 on the cell cycle should be performed.

## 5. Conclusions

In summary, we confirmed that DIAPH1 is overexpressed in LSCC and is involved in the regulation of apoptosis in AMC-HN-8 and FD-LSC-1 cells via the ATR/p53/caspase-3 signaling pathway. Therefore, DIAPH1 may act as an oncogene in LSCC and may be developed as a diagnostic biomarker for, and potential therapeutic target of, LSCC.

## Figures and Tables

**Table 1:** The target sequences of DIAPH1-interfered shRNA.

Name	Target sequence (5′-3′)
DIAPH1-SH1	GGACAAAGGTGAAGGAGGA
DIAPH1-SH2	GGAGAAATCTGAAGCCAAA
DIAPH1-SH3	TCAAGAAGGTGGAGAAGAA
DIAPH1-SH4	GCGAGCAAGTGGAGAATAT

**Table 2:** The primers used for RT-qPCR.

Primer name	Primer sequence (5′-3′)
RPL13A-F	CGAGGTTGGCTGGAAGTACC
RPL13A-R	CTTCTCGGCCTGTTTCCGTAG
DIAPH1-F	GGAGTTACGATAGCCGGAACA
DIAPH1-R	CTTCTGTCTCCAACATGGTCTTG
GAPDH-F	CAAGGTCATCCATGACAACTTTG
GAPDH-R	GTCCACCACCCTGTTGCTGTAG

**Table 3:** The correlation between DIAPH1 expression and clinicopathological characteristics.

Clinicopathological factors	Patients (*N* = 36) *n* (%)	IHS mean rank	*p* value
Age			0.252
<62	20 (55.56)	16.78	
≥62	16 (44.44)	20.66	
T stage			0.173
T1–T2	10 (27.78)	14.80	
T3–T4	26 (72.22)	19.92	
Clinical stage			0.016
I–II	7 (19.44)	10.21	
III–IV	29 (80.56)	20.50	
Lymph node stage			0.889
N0	24 (66.67)	18.33	
N+ (N1 & N2 & N3)	12 (33.33)	18.83	
Primary location			0.841
Supraglottic	21 (58.33)	18.79	
Glottic and subglottic	15 (41.67)	18.10	

## Data Availability

The data used to support the findings of this study are available from the corresponding author upon request. But the corresponding author cannot provide the clinical information of the patients because the information is restricted by the Ethics Committee of Fudan University in order to protect patient privacy. RNA-sequencing analysis of AMC-HN-8 NC and SH1 cells also cannot be provided because further research based on the analysis is being carried out to verify the role of DIAPH1 in laryngeal squamous cell carcinoma.

## Supplementary Materials

Supplementary Materials Figure 1: p53 detection via dna electrophoresis: (1 and 6) markers; (2–3) AMC-HN-8 cells; (4–5) FD-LSC-1 cells. The primers used were as follows: p53 forward: 5′- CCTCAGCATCTTATCCGAGTGG-3′; reverse: 5′- TGGATGGTGGTACAGTCAGAGC-3′.
