# Supplementary material for: DIAPH1 Is Upregulated and Inhibits Cell Apoptosis through ATR/p53/Caspase-3 Signaling Pathway in Laryngeal Squamous Cell Carcinoma
Source: Dis Markers. 2019 Jan 14;2019:6716472. doi: 10.1155/2019/6716472 (PMC6348834; doi:10.1155/2019/6716472)
Supplement: Supplementary Materials — Figure 1: p53 detection via dna electrophoresis: (1 and 6) markers; (2–3) AMC-HN-8 cells; (4–5) FD-LSC-1 cells. The primers used were as follows: p53 forward: 5′- CCTCAGCATCTTATCCGAGTGG-3′; reverse: 5′- TGGATGGTGGTACAGTCAGAGC-3′. [file 6716472.f1.docx]

**Figure Supplementary**
